# Multifunctional Dermatological Effects of Whole-Plant *Bassia scoparia* Extract: Skin Repair and Protection

**DOI:** 10.3390/cimb47080617

**Published:** 2025-08-04

**Authors:** Seogyun Jeong, Hye-Been Kim, Dong-Geol Lee, Eunjin Park, Seoyeon Kyung, Seunghyun Kang, Dayeon Roo, Sang Hyun Moh, Sung Joo Jang, Jihyeon Jang, HyungWoo Jo, Sanghun Lee

**Affiliations:** 1 Department of Bioconvergence and Engineering, Graduate School, Dankook University, Yongin 16890, Republic of Korea; wjdtjrbs2015@naver.com; 2 Research and Innovation Center, Cosmax BTI, Seongnam 13486, Republic of Korea; kimhb@cosmax.com (H.-B.K.); leedg@cosmax.com (D.-G.L.);; 3 Plant Cell Research Institute of BIO-FD&C Co., Ltd., Incheon 21990, Republic of Korea

**Keywords:** whole-plant *Bassia scoparia*, anti-photoaging, anti-inflammatory, anti-angiogenic, wound healing, skin hydration

## Abstract

*Bassia scoparia* (Syn. *Kochia scoparia* (L.) *Schrad.*) is a medicinal plant whose fruit, Kochiae Fructus, has been extensively studied for its dermatological applications. This study focused on extracts from the whole plant *B. scoparia* (WPBS), excluding fruits, to address the research gap regarding the medicinal properties of non-fruit parts. The diverse skin benefits of WPBS, including its anti-photoaging, moisturizing, wound healing, anti-inflammatory, and anti-angiogenic effects, were investigated. The WPBS extract enhanced the viability of keratinocytes (HaCaT) without inducing cytotoxic effects. WPBS significantly reduced *matrix metalloproteinase-1* (*MMP-1*) levels and increased *collagen type I alpha 1* (*COL1A1*) levels (*p* < 0.01) in fibroblasts exposed to ultraviolet B (UVB) radiation, indicating strong anti-photoaging effects. WPBS upregulated skin hydration markers such as *aquaporin-3* (*AQP3*) and *hyaluronan synthase-3* (*HAS3*) and effectively accelerated fibroblast wound closure compared to the positive control. Furthermore, WPBS substantially downregulated the expression of inflammatory (*COX-2* and *IL-1β)* and angiogenic markers (*VEGF)*. Transcriptome analysis (RNA-seq) confirmed that WPBS suppressed inflammation-related and UV-induced gene expression pathways. Overall, these findings expand the therapeutic scope of *B. scoparia* beyond its traditional fruit use and suggest that WPBS is a promising botanical ingredient for various skin applications.

## 1. Introduction

The fruits of *Bassia scoparia* (syn. *Kochia scoparia* (L.) *Schrad.*) are known as Kochiae Fructus (KF) in East Asian traditional medicine [1,2,3]. KF has been used to treat various skin conditions, underscoring its long-standing role in skin care [4]. Consistent with its traditional applications, modern scientific studies have substantiated the therapeutic efficacy of KF and elucidated its molecular mechanisms of action. Notably, KF demonstrated strong anti-photoaging effects in skin models subjected to different ultraviolet (UV) radiation [5,6]. Mechanistically, KF has been shown to enhance transforming growth factor-beta (TGF-β) signaling, which stimulates the production of procollagen 1 while concurrently suppressing matrix metalloproteinase-13 (MMP-13), thereby promoting collagen synthesis and preventing its degradation [5,7]. In addition to their anti-photoaging properties, KF exhibit remarkable anti-inflammatory effects [8]. For example, KF extracts inhibited lipopolysaccharide (LPS)-induced production of nitric oxide (NO), prostaglandin E2 (PGE2), and tumor necrosis factor-α (TNF-α) in RAW 264.7 macrophages by downregulating inducible NO synthase (iNOS) and cyclooxygenase-2 (COX-2) expression at the mRNA and protein levels [9]. KF has also been reported to inhibit angiogenesis, likely by downregulating PI3K/AKT/mTOR signaling and reducing the phosphorylation of vascular endothelial growth factor (VEGF) receptor 2 [10]. The anti-inflammatory and anti-angiogenic activities of KF are believed to contribute to its effectiveness in alleviating erythema and other inflammatory skin conditions [11]. KF may additionally possess wound-healing properties [8]. In contact dermatitis models, topical application of KF methanol extracts significantly reduced inflammation and effectively inhibited epidermal spongiosis and intercellular edema. Since inflammation control is crucial for optimal wound healing, these anti-inflammatory effects suggest that KF can facilitate tissue repair by creating a favorable healing environment.

Despite these promising findings, research on *B. scoparia* has predominantly focused exclusively on KF, leaving the potential of other parts of the plant largely unexplored. Studies on other medicinal species (e.g., *Balanites aegyptiaca* and *Schisandra chinensis*) have shown that non-fruit parts (leaves and stems) possess significantly different metabolite profiles and therapeutic properties than fruits [12,13]. In some cases, extracts from the entire plant (e.g., *Momordica foetida* and *Fragaria vesca*) exhibit superior biological activities compared to fruit extracts [14,15]. Plants contain multiple bioactive compounds that exert diverse physiological effects via different mechanisms of action [16]. Focusing solely on individual biological effects may limit our understanding of the plant’s therapeutic potential. In contrast, a comprehensive evaluation can reveal the simultaneous presence of multiple beneficial properties, such as anti-inflammatory and wound-healing activities. Scientific research demonstrates that several plants exhibit multifunctional properties by targeting multiple biological processes simultaneously [17,18]. This multitarget nature supports the need for a comprehensive assessment of multiple biological activities rather than focusing on individual effects. Collectively, these observations suggest that whole plant *B. scoparia* (WPBS), excluding fruits, may possess unexplored therapeutic potential for dermatological applications, which represents a significant knowledge gap.

To the best of our knowledge, no experimental study has investigated the dermatological effects of WPBS, excluding fruits. Therefore, our study aimed to comprehensively evaluate the various skin-related biological activities of WPBS, including its anti-photoaging, moisturizing, wound healing, anti-inflammatory, and anti-angiogenic effects. This integrated approach expands the therapeutic understanding and potential applications of *B. scoparia* beyond traditional fruit-based uses.

## 2. Materials and Methods

### 2.1. Preparation of WPBS Extracts

**Figure 1:**
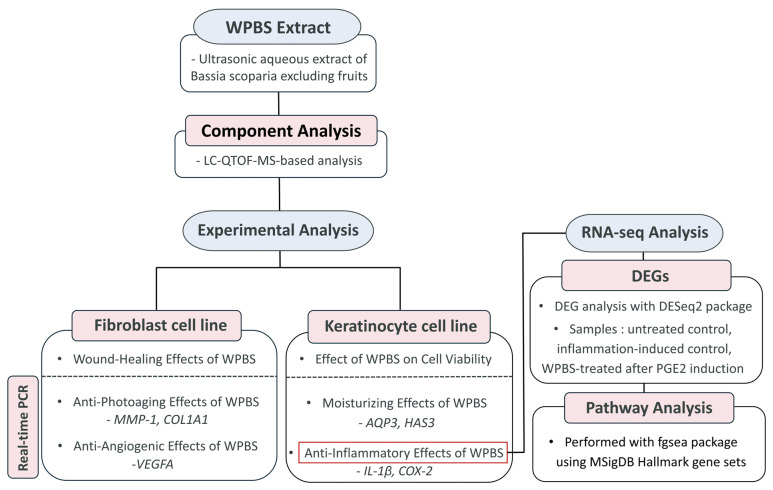
Experimental workflow and study design for WPBS biological evaluation. WPBS extract was prepared via ultrasonic aqueous extraction of Bassia scoparia excluding fruits, followed by LC-QTOF-MS component analysis. Biological evaluation was conducted using keratinocyte and fibroblast cell lines to assess dermatological effects via real-time PCR. RNA-seq analysis with DEG identification and pathway analysis was performed using DESeq2 and fgsea packages. WPBS, whole plant *B. scoparia*; DEGs, differentially expressed genes.

The experimental workflow of the WPBS extract preparation for biological evaluation is illustrated in Figure 1. An aqueous WPBS extract was prepared from dried *Bassia scoparia* plants (excluding fruits). The dried plants were pulverized, and 5 g of the powdered plant material was mixed with 5 L of purified water and subjected to ultrasonic extraction for 2 h at 40 kHz and 700 W (PowerSonic 520, Hwashin Technology, Seoul, Republic of Korea). The extract was filtered and concentrated for use in the subsequent experiments.

### 2.2. LC-QTOF-MS Analyses

Liquid chromatography-quadrupole time-of-flight-mass spectrometry (LC-QTOF-MS) was performed using an Agilent 6546 LC/QTOF system with MassHunter software (v10.0). The injection volume was 4 µL at 25 °C. The mobile phases used were solvent A (water with 10 mM ammonium formate and 0.1% formic acid) and solvent B (90% acetonitrile with 10 mM ammonium formate and 0.1% formic acid). The gradient was increased from 2% solvent A to 95% solvent A over 16 min. Mass spectrometry used a dual AJS ESI source at 225 °C with a nebulizer pressure of 40 psi and capillary voltage of 3000 V.

### 2.3. Cell Line and Cell Culture (Keratinocyte HaCaT)

The HaCaT human keratinocyte cell line was obtained from the American Type Culture Collection (ATCC, Manassas, VA, USA) and maintained in Dulbecco’s Modified Eagle’s medium (DMEM; HyClone, Logan, UT, USA) supplemented with 10% fetal bovine serum (FBS) and 1% antibiotic–antimycotic solution. The cells were cultured at 37 °C in a humidified incubator with 5% CO_2_ and used for experiments at approximately 80% confluence. Prior to treatment, the HaCaT cells were incubated with menthol (100 μM) in serum-free medium for 24 h to induce a controlled stress response.

#### 2.3.1. Cell Viability Assay

HaCaT cells were exposed to various concentrations of WPBS (1, 5, and 10%) in serum-free DMEM for 24 h. Cell viability was assessed by adding 20 μL of 3-(4,5-dimethyl-2-thiazolyl)-2,5-diphenyl-2H-tetrazolium bromide (MTT) solution (5000 μg/mL) to each well, and the plates were incubated for 4 h. Following incubation, the supernatant was removed, the formazan crystals were dissolved in 100 μL of dimethyl sulfoxide (DMSO), and cell viability was assessed by comparing the absorbance measured at 540 nm using a microplate reader relative to the untreated controls.

#### 2.3.2. Moisturizing Model and Treatment

To evaluate the effects on skin hydration markers, HaCaT cells were treated with WPBS extract (1% or 10%) or 1 μM retinoic acid (RA) as a positive control in serum-free medium for 24 h. RA enhances aquaporin-3 (AQP3) expression and its biological activity, promoting glycerol influx into skin cells and improving skin hydration and function [19,20]. After treatment, the cells were harvested to analyze the expression of hydration-related genes, including AQP3 and hyaluronan synthase-3 (HAS3) [21].

#### 2.3.3. Inflammatory Model and Treatment

HaCaT cells were stimulated with 10 μg/mL polyinosinic-polycytidylic acid (poly I:C) and 10 ng/mL IL-4 in the presence of 1% antibiotic–antimycotic to mimic an inflammatory environment [22]. After 24 h of stimulation, WPBS extract (1%) or anti-inflammatory dexamethasone (1 μM; Sigma-Aldrich, St. Louis, MO, USA) was added to the cells in serum-free medium and incubated for 4 h. Dexamethasone, known to inhibit the activation of the toll-like receptor 3 (TLR3) signaling pathway by poly I:C, thereby suppressing inflammation and reducing the release of pro-inflammatory cytokines, was used as a positive control [23]. Cells were then collected for the analysis of inflammatory gene expression (e.g., IL1B and PTGS2 corresponding to COX-2).

### 2.4. Fibroblast (Hs68) Cell Culture for Treatment

The human dermal fibroblast cell line Hs68 (ATCC, Manassas, VA, USA) was cultured in DMEM supplemented with 10% FBS and 1% antibiotic–antimycotic at 37 °C in a humidified incubator with 5% CO_2_. Cells were seeded in 6-well plates at approximately 80% confluence and incubated for 24 h prior to treatment.

#### 2.4.1. UVB-Induced Photoaging Model and Treatment

To model photoaging, confluent Hs68 fibroblasts in 6-well plates were rinsed with PBS and exposed to UVB irradiation at 15 mJ/cm^2^ (wavelength range: 290–320 nm; peak at 311 nm) using a UV crosslinker (UVP; Upland, CA, USA). Immediately after UVS exposure, the cells were treated with WPBS extract (1% and 10%) or RA (1 μM) in serum-free DMEM and incubated for 24 h. RA was used as a positive control because of its known protective effect against UVB-induced skin damage [24]. After 24 h, the cells were harvested for analysis of photoaging-related genes, such as *matrix metalloproteinase-1 (MMP-1)* and *collagen type I alpha 1 chain (COL1A1)* [25].

#### 2.4.2. Wound-Healing (Migration) Assay

Fibroblast wound healing was assessed using the scratch migration assay. Hs68 cells (2 × 10^4^ cells/well) were seeded in Incucyte^®^ Imagelock 96-well plates (Essen Bioscience, Ann Arbor, MI, USA) at approximately 90% confluence and incubated overnight at 37 °C in a humidified 5% CO_2_ environment. A uniform scratch wound was created in each well using the IncuCyte^®^ Maker tool (Essen Bioscience). The cells were then treated with the WPBS extract (1% and 10%) in FBS-free DMEM. The control wells received no treatment (serum-free medium only), and the positive control wells were treated with 10% FBS (a potent stimulator of cell migration). Plates were placed in an Incucyte^®^ Live-Cell Analysis System (Essen Bioscience) prewarmed to 37 °C for 30 min prior to scanning. Wound closure was monitored by capturing digitized images of the culture fields every 2 h for up to 24 h after scratching. Images were captured and analyzed using the Incucyte^®^ Live-Cell Analysis System. The wound area in each image was quantified to calculate the percentage wound closure over time.

#### 2.4.3. Anti-Angiogenic Model and Treatment

To examine the effects of angiogenesis-related factors, Hs68 fibroblasts were stimulated with 30 nM PGE2 (Sigma-Aldrich) for 1 h to induce the expression of pro-angiogenic genes (notably VEGFA). After stimulation, cells were treated with WPBS (1% and 10%) or a positive control, ceramide 3B (30 μM; GLPBIO, Montclair, NJ, USA), in serum-free medium and incubated for 72 h. Ceramide 3B was selected as a positive control because ceramide signaling inhibits angiogenesis by inducing dephosphorylation of key signaling enzymes [26]. After treatment, fibroblasts were collected to assess VEGFA mRNA levels.

### 2.5. RNA Extraction and Real-Time PCR

Total RNA was extracted from the cells using TRIzol reagent (Takara, Shiga, Japan), according to the manufacturer’s instructions. The residual RNA was further purified using an RNeasy Mini Kit (Qiagen, Hilden, Germany). Complementary DNA (cDNA) was synthesized from 1 μg of total RNA using reverse transcription premix (Elpis-Biotech, Daejeon, Republic of Korea) under the following reaction conditions: 45 °C for 45 min and 95 °C for 5 min. Quantitative real-time PCR (qPCR) was performed using a StepOne Plus™ System Software (v2.3; Applied Biosystems, Foster City, CA, USA) with SYBR Green PCR Master Mix (Applied Biosystems) according to the manufacturer’s protocol. Gene-specific primers were used in the ABI 7300 system (Bioneer, Daejeon, Republic of Korea): β-actin (F: 5′-GGCCATCTCTTGCTCGAAGT-3′ and R: 5′-GACACCTTCAACACCCAGC-3′), AQP3 (F: 5′-GTCACTCTGGGCATCCTCAT-3′ and R: 5′-CTATTCCAGCACCCAAGAAGG-3′), HAS3 (F: 5′-CTTAAGGGTTGCTTGCTTGC-3′ and R: 5′-GTTCGTGGGAGATGAAGGAA-3′), IL-1β (F: 5′-GTCATTCGCTCCCACATTCT-3′ and R: 5′-ACTTCTTGCCCCCTTTGAAT-3′), COX-2 (F: 5′-TTCAAATGAGATTGTGGGAAAAT-3′ and R: 5′-AGATCATCTCTGCCTGAGTATCTT-3′), MMP-1 (F: 5′-CGAAATTTGCCGACAGAGATGA-3′ and R: 5′-GTCCCTGAACAGCCCAGTACTT-3′), and COL1A1 (F: 5′-CTCGAGGTGGACACCACCCT-3′ and R: 5′-CAGCTGGATGGCCACATCGG-3′) and VEGF-A (F: 5′-AGGGCAGAATCATCACGAAGT-3′ and R: 5′-AGGGTCTCGATTGGATGGCA-3′). The thermal cycling conditions were set as follows: initial activation at 50 °C for 2 min and 95 °C for 10 min, followed by 40 amplification cycles at 95 °C for 10 s and 60 °C for 1 min. β-Actin was used as an internal control for normalization.

### 2.6. RNA Sequencing and Bioinformatic Analysis

#### 2.6.1. Sample Preparation for RNA-Seq

The flowchart and scripts used for bioinformatics analysis are presented in Figure S2 and File S1, respectively. Raw RNA-seq data were deposited in Zenodo and are available at https://doi.org/10.5281/zenodo.15780950.

For transcriptomic analysis, Hs68 fibroblasts were seeded at a density of 1 × 10^4^ cells/mL in 6-well culture plates and initially incubated for 72 h at 37 °C in a humidified atmosphere containing 5% CO_2_. After reaching the approximate proliferative stage, as visually assessed under a microscope, the culture medium was replaced, and 30 nM PGE2 was added to induce capillary expansion. Subsequently, WPBS extract was administered at concentrations of 10 and 100 μg/mL, and cells were incubated for an additional 72 h under the same conditions. At the end of the incubation period (144 h after the initial seeding), the cells were harvested, and total RNA was extracted under three conditions: (1) untreated control (NC), (2) inflammation-induced control (PGE2-treated), and (3) WPBS-treated cells after PGE2 induction. High-quality RNA (RIN > 7) was obtained using the QIAzol Lysis Reagent and RNeasy Mini Kit (Qiagen, Hilden, Germany).

#### 2.6.2. Library Construction and Sequencing

RNA sequencing libraries were constructed using the TruSeq Stranded mRNA Sample Preparation Kit (Qiagen, Hilden, Germany). In brief, poly-A mRNA was isolated from 1 μg of total RNA per sample using oligo (dT) magnetic beads and then fragmented at a high temperature. First-strand cDNA was synthesized using SuperScript II reverse transcriptase (Invitrogen, Carlsbad, CA, USA) and random primers, followed by second-strand cDNA synthesis using DNA Polymerase I and RNase H. Double-stranded cDNA fragments were end-repaired, A-tailed, and ligated to the indexed adaptors. The libraries were PCR-amplified and their quality was assessed using an Agilent TapeStation D1000 ScreenTape system. Sequencing was performed using the Illumina NovaSeq X platform (Illumina, San Diego, CA, USA) to generate 2 × 151 bp paired-end reads.

#### 2.6.3. Differential Gene Expression and Pathway Analysis

Raw sequencing reads were initially assessed using FastQC software (version 0.11.9), and adapter sequences were subsequently trimmed using Cutadapt (version 4.6) [27,28]. The cleaned reads were aligned to the human reference genome (GRCh38) using HISAT2 (version 2.2.1) [29] and gene-level counts were obtained using featureCount (version 2.0.8) [30]. Differentially expressed genes (DEGs) between groups were identified using the DESeq2 package (version 1.46.0) [31] with a significance criterion of FDR q-value < 0.05, and an absolute log_2_ fold change > 1. DEGs were visualized using volcano plots and heat maps to illustrate changes in expression. Gene set enrichment analysis (GSEA) was performed using the fgsea package (version 1.34.0) on the MSigDB Hallmark gene sets to identify enriched pathways [32,33]. Pathways with an FDR q-value < 0.05 were considered significantly enriched.

### 2.7. Statistical Analysis

Each experiment was conducted at least three times. The results are presented as the mean ± standard deviation. For in vitro cell assay analysis, statistical evaluations were performed using the SPSS statistical package (version 25.0; SPSS Inc., Chicago, IL, USA). Statistical comparisons between the two groups were performed using a two-tailed Student’s *t*-test. In all tests, *p* < 0.05 was deemed statistically significant, while *p* < 0.01 and *p* < 0.001 were considered highly significant.

## 3. Results

### 3.1. Identification of Bioactive Compounds in WPBS Extract

**Figure 2:**
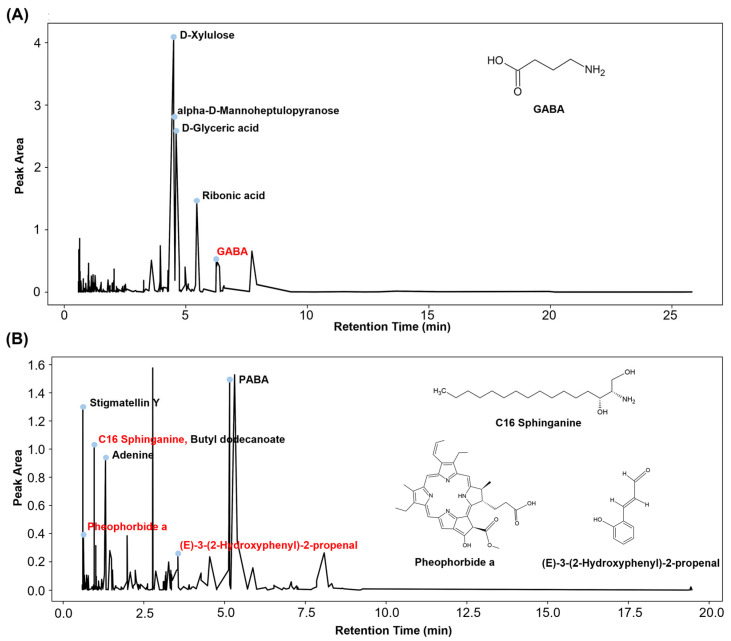
Bioactive compounds in whole-plant *B. scoparia* (WPBS) extract. The *X*-axis represents retention time (minutes) and the *Y*-axis represents peak area as relative abundance. (**A**) Negative ion mode analysis identified major compounds including D-xylulose, alpha-D-mannoheptulopyrranose, D-glyceric acid, ribonic acid, and gamma-aminobutyric acid (GABA). (**B**) Positive ion mode analysis revealed a high abundance of stigmatellin Y and 4-aminobenzoic acid (PABA), along with C16 sphinganine, adenine, butyl dodecanoate, pheophorbide a, and (E)-3-(2-hydroxyphenyl)-2-propenal. Compounds with known anti-photoaging, moisturizing, wound-healing, anti-inflammatory, and anti-angiogenic properties are highlighted in red text, and their chemical structures are shown on the right.

LC-QTOF-MS analysis revealed diverse bioactive compounds in the WPBS extract; non-relevant or non-plant-derived compounds were excluded from the analysis (Figure 2 and Table S1). The negative ion mode (Figure 2A) primarily detected d-xylulose, alpha-d-mannoheptulopyranose, d-glyceric acid, and ribonic acid, along with bioactive compounds such as gamma-aminobutyric acid (GABA; PubChem CID: 119). Positive-ion mode analysis (Figure 2B) revealed a high abundance of stigmatellin Y and 4-aminobenzoic acid (PABA). Additionally, several bioactive compounds, including C16 sphinganine, adenine, butyl dodecanoate, pheophorbide a, and (E)-3-(2-hydroxyphenyl)-2-propenal, were identified. Among these, GABA [34], C16 sphinganine (CID: 656816) [35], pheophorbide a (CID: 253193) [36], and (E)-3-(2-hydroxyphenyl)-2-propenal (CID: 5318169) [37] are known for their anti-photoaging, moisturizing, wound-healing, anti-inflammatory, and anti-angiogenic effects, indicating potential therapeutic applications of WPBS extract.

### 3.2. Effect of WPBS on Cell Viability

WPBS treatment enhanced the keratinocyte viability without inducing cytotoxicity (Figure S1). After 24 h of treatment, HaCaT cells showed approximately 23%, 25%, and 18% higher viability in 1%, 5%, and 10% WPBS, respectively, compared to untreated controls. Even at the highest concentration (10%), WPBS did not reduce cell viability to below the control levels, indicating a lack of toxicity. These results confirm that WPBS has no adverse effects on skin cells and can modestly enhance cell viability.

### 3.3. Anti-Photoaging Effects of WPBS

**Figure 3:**
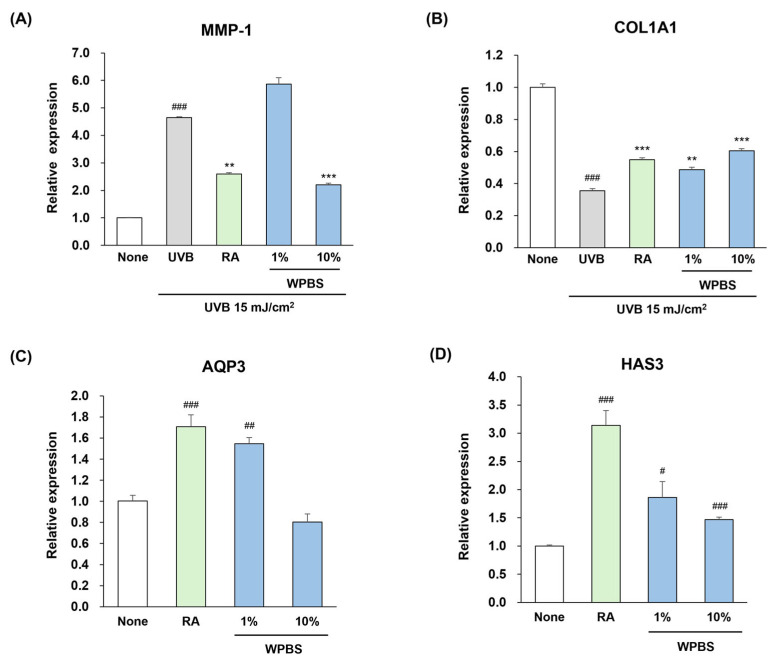
Protective effects of whole-plant *B. scoparia* (WPBS) extract against UVB-induced photoaging and skin hydration markers in cell culture. (**A**) Hs68 fibroblasts exposed to UVB irradiation (15 mJ/cm^2^) and MMP-1 mRNA expression compared to the non-irradiated control (None). A significant reduction in UVB-induced MMP-1 expression by WPBS treatment (10%) to a level comparable or slightly superior to that of the positive control, retinoic acid (RA, 1 μM). (**B**) UVB exposure induced a decrease in Collagen Type I Alpha 1 chain (COL1A1) mRNA in fibroblasts and significantly restored COL1A1 expression by WPBS (1% and 10%), with efficacy similar to RA. (**C**) Significantly upregulated aquaporin-3 (AQP3) mRNA in HaCaT keratinocytes treated with WPBS (1%) to a level comparable with RA (*p* < 0.01); no effect of 10% WPBS. (**D**) WPBS (1% and 10%) increased HAS3 mRNA expression with a greater effect at 1%. Statistical significance: # *p* < 0.05, ## *p* < 0.01, ### *p* < 0.001 vs. none; ** *p* < 0.01, *** *p* < 0.001 vs. UVB.

The effects of WPBS on cell photoaging were evaluated by measuring the expression levels of *MMP-1* and *COL1A1* in UVB-irradiated fibroblasts (Hs68). In this model, WPBS exerted potent anti-photoaging effects (Figure 3). Specifically, 10% WPBS significantly downregulated *MMP-1* expression compared to that in the UV-only control group (*p* < 0.001; ***, Figure 3A) and simultaneously upregulated *COL1A1* expression (*p* < 0.001; ***, Figure 3B). The magnitude of these effects with 10% WPBS was slightly greater than that observed with the positive control RA (1 μM). Thus, WPBS effectively shielded fibroblasts from UVB-induced collagen degradation, indicating its potential to prevent UV-induced skin aging by maintaining the expression of collagen-related genes.

### 3.4. Moisturizing Effects of WPBS

To assess whether WPBS improves the expression of skin hydration factors, we measured the mRNA levels of *AQP3* and *HAS3* in HaCaT keratinocytes after treatment. The group treated with 1% WPBS exhibited upregulation of *AQP3* expression compared to untreated cells, reaching levels comparable to those induced by RA (1 μM), whereas 10% WPBS treatment did not enhance AQP3 expression (Figure 3C). Both 1% and 10% WPBS treatments elevated *HAS3* expression, with the 1% concentration causing a greater increase than the 10% concentration (Figure 3D). These findings indicate that WPBS, particularly at lower concentrations, can upregulate the key genes involved in epidermal hydration, implying a beneficial moisturizing effect on the skin.

### 3.5. Wound-Healing Effects of WPBS

**Figure 4:**
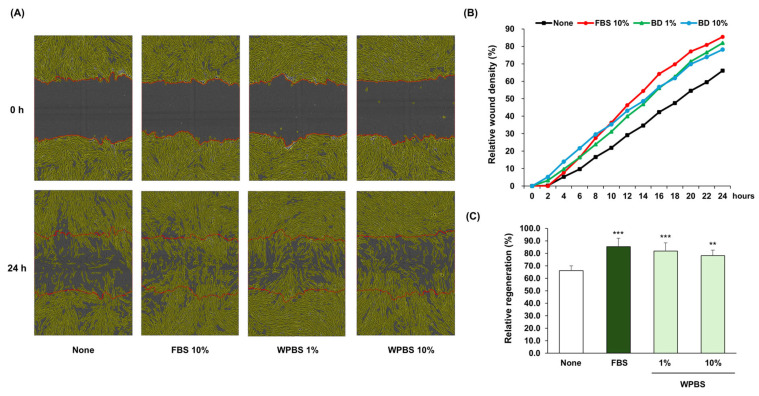
Wound-healing efficacy of WPBS in Hs68 fibroblasts. (**A**) Scratch-wound assay images (0 and 24 h post-wounding) showing substantially greater wound closure in fibroblasts treated with WPBS (1% or 10%) compared with that in untreated controls and comparable to that in cells treated with 10% FBS (positive control). (**B**) Time-course analysis of wound closure over 24 h showing that WPBS (1% and 10%) accelerated wound healing rates, closely approaching healing in the 10% FBS-treated positive control. (**C**) Quantification of wound closure 24 h post-scratch showing that WPBS significantly enhanced wound regeneration compared to untreated cells (~80% closure vs. ~50% in controls). Healing in the 1% WPBS-treated group was slightly better than in the 10% WPBS-treated group. Statistical significance: ** *p* < 0.01, *** *p* < 0.001, vs. none.

The ability of WPBS to facilitate wound healing was assessed using the fibroblast scratch assay. After 24 h, untreated control cells achieved only partial wound closure, whereas cultures treated with 1% or 10% WPBS exhibited complete wound closure, comparable to the healing observed in the positive control (10% FBS) (Figure 4A). Analysis of wound closure dynamics using time-lapse imaging showed that 1% and 10% WPBS markedly accelerated wound coverage compared with untreated cells (Figure 4B). At 24 h post-wounding, the relative wound closure was ~82% in the 1% WPBS-treated group and ~78% in the 10% WPBS-treated group versus a much lower closure in the controls (Figure 4C). These data demonstrated that WPBS promotes fibroblast migration and proliferation, thereby effectively accelerating wound healing in vitro.

### 3.6. Anti-Inflammatory and Anti-Angiogenic Effects of WPBS

**Figure 5:**
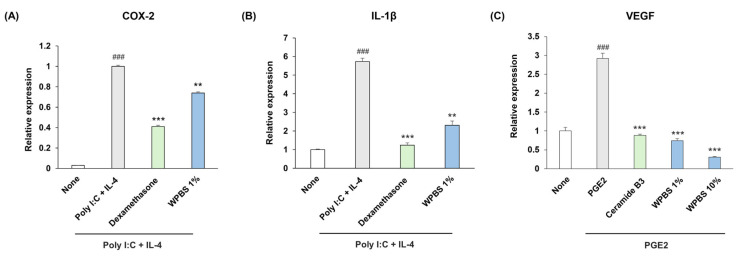
Anti-inflammatory and anti-angiogenic effects of WPBS. (**A**) Poly (I:C) + IL-4 induced inflammation increased *COX-2* mRNA levels in HaCaT keratinocytes. WPBS (1%) treatment significantly decreased *COX-2* expression compared with the stimulated control, but not as strongly as dexamethasone (1 μM, positive control). (**B**) Increased *IL-1β* mRNA in HaCaT cells following inflammation induced by poly (I:C) + IL-4 significantly reduced *IL-1β* expression induced by WPBS (1%). (**C**) Elevated *VEGF* mRNA levels in Hs68 fibroblasts following PGE2 stimulation (30 nM). VEGF expression was downregulated by WPBS (1% and 10%) relative to PGE2-only treatment, with efficacy comparable to or exceeding that of the positive control, ceramide 3 B (30 μM, *p* < 0.001). Statistical significance: ^###^ *p* < 0.001 vs. none; ** *p* < 0.01, *** *p* < 0.001 vs. PGE2-treated control.

We assessed the anti-inflammatory effects of WPBS on HaCaT keratinocytes and its antiangiogenic effects on Hs68 fibroblasts, respectively. In the keratinocyte inflammation model, stimulation with poly (I:C) + IL-4 strongly induced *COX-2* and *IL-1β* expression (as expected), whereas treatment with dexamethasone notably suppressed these genes, validating the model. The expression of *COX-2* was reduced, and that of *IL-1β* was significantly decreased by 1% WPBS treatment compared to that in the stimulated control (*p* < 0.01; ** in Figure 5A,B), although WPBS was less effective than dexamethasone.

In a fibroblast angiogenesis model, PGE2 stimulation significantly increased in *VEGF* expression. WPBS (1%) markedly downregulated *VEGF* mRNA compared to the PGE2-only condition, with WPBS (10%) demonstrating an effectiveness comparable to or slightly exceeding that of the positive control, ceramide 3 B (*p* < 0.001; *** in Figure 5C). Collectively, these results indicate that WPBS exhibits strong anti-inflammatory properties (by limiting the induction of pro-inflammatory cytokines and enzymes) and antiangiogenic activity (by inhibiting *VEGF* expression).

### 3.7. RNA-Seq-Based Transcriptomic Analysis of WPBS Effects

**Figure 6:**
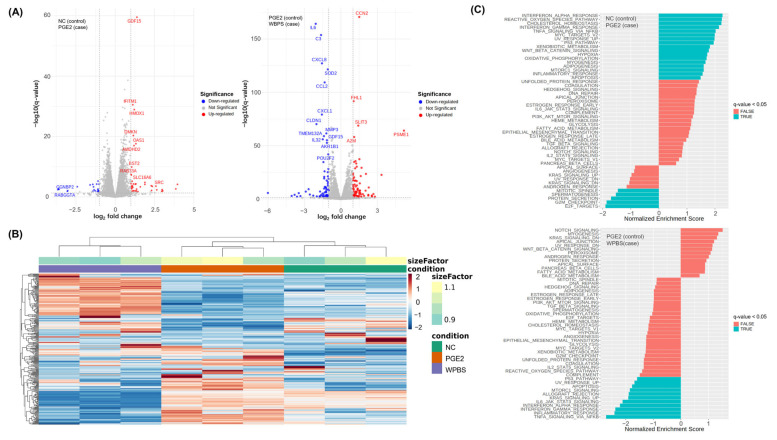
RNA-seq analysis of the effect of WPBS treatment under inflammatory conditions. (**A**) Volcano plot of the changes in gene expression. Left: Inflammation (PGE2) vs. control (NC); several genes (red) upregulated by PGE2 (e.g., *GDF15*, *IFITM1*, and *HMOX1*) confirmed the induction of inflammation. Right: WPBS-treated (after PGE2) vs. PGE2-only; numerous pro-inflammatory genes (blue) were downregulated by WPBS (e.g., *IL6*, *CXCL8*, and *C3*). Dotted lines indicate significance thresholds (q-values < 0.05). (**B**) Heatmap of global gene expression (top 50 DEGs) shows distinct clustering of samples: WPBS-treated cells clustered separately from PGE2-only cells and closer to untreated controls. (**C**) GSEA of Hallmark pathways. Top: In PGE2 vs. control, inflammatory and stress pathways (“INTERFERON_ALPHA_RESPONSE,” “UV_RESPONSE_UP,” “INFLAMMATORY_RESPONSE”) were positively enriched in PGE2-treated cells. Bottom: In WPBS vs. PGE2, the same pathways (including “TNFA_SIGNALING_VIA_NFKB”) were negatively enriched with WPBS treatment (q-value < 0.05), indicating that WPBS suppressed these pathways.

To gain a more comprehensive understanding of the mechanisms underlying the effects of WPBS, we performed RNA-seq on fibroblasts under NC, inflammatory (PGE2-stimulated), and WPBS-treated (post-PGE2 stimulation) conditions. The transcriptomic data were consistent with the functional observations. Analysis using volcano plots revealed that PGE2 stimulation (vs. NC) significantly upregulated multiple inflammation-related genes, such as *growth differentiation factor 15 (GDF15)*, *interferon-induced transmembrane protein 1* (*IFITM1)*, and *heme oxygenase 1* (*HMOX1)* (Figure 6A, left), confirming the successful induction of an inflammatory state.

In contrast, WPBS treatment after PGE2 exposure (vs. PGE2 alone) significantly downregulated of several pro-inflammatory genes, including *interleukin 6 (IL6)*, *complement C3 (C3)*, and *interleukin-8 (CXCL8).* This suggested that WPBS effectively reduced the inflammatory response at the transcriptional level (Figure 5A, right panel). A heatmap illustrating global gene expression distinctly separated the three groups (NC, PGE2, and WPBS), with WPBS-treated samples forming a separate cluster from those treated only with PGE2 (Figure 6B). GSEA further reinforced these observations: hallmark pathways such as “INTERFERON_ALPHA_RESPONSE,” “UV_RESPONSE_UP,” and “INFLAMMATORY_RESPONSE” were significantly enriched in the PGE2-treated group compared to control (q-value < 0.05), whereas these same pathways (including “TNFA_SIGNALING_VIA_NFKB”) were significantly negatively enriched in WPBS-treated cells relative to the PGE2 group (q-value < 0.05) (Figure 6C). This indicates that WPBS broadly suppresses inflammatory and stress-response signaling induced by PGE2. The GSEA results for the hallmark and additional Gene Ontology and Reactome databases are presented in Table S2. Overall, the RNA-seq analysis validated the dual anti-inflammatory and photoprotective actions of WPBS at the molecular level.

## 4. Discussion

This study provides novel insights into the dermatological potential of WPBS, demonstrating that whole *B. scoparia* extracts (excluding the fruit) confer anti-photoaging, moisturizing, wound-healing, anti-inflammatory, and anti-angiogenic effects. By extending these investigations beyond traditionally used fruits, we showed that WPBS can influence the key molecular pathways involved in skin health.

UVB-induced skin aging is primarily caused by the activation of the MAPK pathways (ERK, JNK, and p38) and AP-1 transcription factors (c-Fos/c-Jun), which upregulate MMP-1 and other matrix metalloproteinases that degrade collagen in the dermal extracellular matrix [38,39,40]. In our UVB model, WPBS treatment suppressed *MMP-1 expression* and enhanced *COL1A1* expression, suggesting a dual mechanism of action. This protective effect may be attributed to the presence of pheophorbide a in WPBS, which has been reported to reduce MMP secretion and expression (MMP-1, MMP-2, MMP-9), increase collagen synthesis (COL1A1/COL1A2), and inhibit UVB-induced ERK and JNK phosphorylation and NF-κB p65 activation [36]. These findings suggest that WPBS disrupts the UVB-activated MAPK/AP-1 cascade, thereby mitigating molecular processes (collagen degradation and ECM damage) and resulting in wrinkle formation and decreased skin elasticity.

In addition to photoprotection, WPBS also displayed significant moisturizing activity. *AQP3* and *HAS2* are crucial for maintaining skin hydration, facilitating water/glycerol transport and hyaluronic acid production, respectively [41,42,43]. Marked upregulation of AQP3 and HAS3 was observed in WPBS-treated keratinocytes, indicating improved hydration potential. This effect may be partially attributed to GABA present in WPBS, which upregulates filaggrin and AQP3 expression in keratinocytes, thereby improving skin moisturization [34]. Interestingly, similar upregulation of these genes has been reported for *Artemisia argyi* extracts, which act via EGFR-mediated STAT3/MAPK signaling [44]. Given the traditional dermatological applications and comparable phenotypic outcomes of WPBS and *A. argyi*, WPBS may also exert moisturizing effects through analogous pathways (e.g., activating EGFR→STAT3/MAPK signaling to enhance *AQP3/HAS3* expression), although this remains to be confirmed experimentally.

WPBS significantly enhanced wound healing in vitro as demonstrated by the rapid migration of fibroblasts and wound closure. This wound-healing activity may be attributed to the presence of (E)-3-(2-hydroxyphenyl)-2-propenal (2′-hydroxycinnamaldehyde) identified in WPBS [37]. This cinnamon-derived aldehyde has well-documented anti-inflammatory and wound-healing properties, inhibiting pro-inflammatory mediators (e.g., NO, TNF-α) and thereby accelerating wound repair. Further investigations are needed to elucidate the specific molecular pathways through which WPBS mediates wound healing. The findings of this study highlighted the anti-inflammatory and anti-angiogenic effects of WPBS, which are particularly relevant to inflammatory skin conditions. *COX-2*, *IL-1β*, and *VEGF* play crucial roles in skin inflammation and erythema through interlinked mechanisms. *COX-2* generates PGE2, a vasodilator that enhances blood flow and causes redness and swelling [45,46,47]. *IL-1β* is a key pro-inflammatory cytokine that intensifies inflammatory responses, including the induction of *IL-6* and chemokines, further enhancing vascular permeability [48]. *VEGF* promotes new vessel formation and leakage, contributing to redness and edema in inflamed skin [49]. These factors often create a positive feedback loop that exacerbates conditions, such as dermatitis and rosacea. WPBS treatment effectively reduced COX-2, IL-1β, and VEGF expression, indicating its potential to disrupt the inflammatory cycle. By targeting multiple upstream mediators of inflammation and angiogenesis, WPBS may be an effective therapeutic agent for reducing erythema and vascular inflammation in the skin.

Our transcriptomic data provided a mechanistic framework for understanding the effects of WPBS. The downregulated expression of genes associated with inflammation (e.g., *IL-1β*, *IL6*, *CXCL8*, *CCL2*, and *PTGS2)* and UV stress (*MMP14*, *ICAM1*, *HMOX1*, and *CXCL2)* was consistent with the observed phenotypic effects of WPBS. Notably, this broad-spectrum modulation may be particularly beneficial in postmenopausal women or individuals with estrogen-deficient skin. The menopause-related estrogen decline leads to impaired skin barrier function, reduced collagen levels, and increased vulnerability to ultraviolet (UV) damage and inflammation [50,51]. Consequently, the menopausal skin often undergoes accelerated photoaging and chronic inflammation. The ability of WPBS to enhance hydration, preserve collagen, and suppress inflammatory pathways indicates its potential for development as a targeted anti-aging and anti-inflammatory treatment to improve skin health in menopausal women and other high-risk groups.

Despite the promising efficacy of WPBS, this study has several important limitations that should be addressed in future research. First, we examined changes in gene expression at the mRNA level using RT-PCR and RNA-seq but did not confirm all corresponding protein-level changes. While acknowledging that post-transcriptional regulation can occur, recent studies have shown that differentially expressed mRNAs correlate significantly better with their protein products than non-differentially expressed mRNAs do.

This finding strongly supports the correlation between the mRNA and protein levels in differential expression studies [52]. This correlation was further confirmed in studies on *B. scoparia* fruit extracts, where transcriptomic results were successfully validated through protein-level analysis, demonstrating consistent anti-photoaging and anti-inflammatory effects [5,53]. Based on this evidence, the RNA-level changes observed in our study are expected to reflect meaningful protein-level effects, and direct protein validation will be planned in future studies.

Secondly, the optimal effective concentration of WPBS varied across different assays; each biological effect (collagen protection and wound healing) was observed at slightly different doses of WPBS. This variability complicates the direct comparison of outcomes and makes it difficult to establish a single optimal dose for all effects. However, this implies that WPBS can be tailored in a dose-dependent manner for specific applications (e.g., lower moisturizing dose vs. higher photoprotective dose). Further dose–response studies in more complex models (3D skin equivalents or animal models) will be valuable for determining the appropriate use and formulation of WPBS-based interventions.

## 5. Conclusions

In conclusion, WPBS demonstrated substantial dermatological efficacy in multiple applications. Specifically, WPBS exhibited significant anti-photoaging effects by downregulating *MMP-1* and upregulating *COL1A1* expression, presenting enhanced efficacy compared with retinoic acid. WPBS also markedly improved wound healing, achieving approximately 80% wound closure within 24 h, compared to approximately 65% in the controls. Additionally, WPBS enhanced moisturizing properties by increasing the expression of hydration-related genes (*AQP3* and *HAS3*), demonstrating effects not previously investigated in KF. Furthermore, WPBS exhibited anti-inflammatory activity by notably reducing *COX-2* and *IL-1β* expression and demonstrated anti-angiogenic effects through effective downregulation of *VEGF* expression. The broad activity profile of WPBS indicates its potential as a targeted botanical therapy for inflammatory, erythematous, and age-associated skin conditions. Importantly, this study extended previous *B. scoparia* research beyond fruit-focused studies to establish the dermatological potential of whole plant biomass. Further in vivo and clinical studies are warranted to validate these effects and to thoroughly investigate the translational potential of WPBS in dermatology.

## Data Availability

The original contributions of this study are included in the article and Supplementary Materials. All RNA-seq data were deposited in Zenodo (https://doi.org/10.5281/zenodo.15780950). Further inquiries can be directed to the corresponding author.

## Supplementary Materials

The following supporting information can be downloaded at: https://www.mdpi.com/article/10.3390/cimb47080617/s1.

## Abbreviations

The following abbreviations are used in the manuscript.

ATCC	American Type Culture Collection
*AQP3*	*Aquaporin-3*
*C3*	*Complement C3*
cDNA	Complementary DNA
*COL1A1*	*collagen type I alpha 1*
COX-2	cyclooxygenase-2
*CXCL8*	*Interleukin-8*
DEGs	differentially expressed genes
DMEM	Dulbecco’s modified Eagle’s medium
DMSO	dimethyl sulfoxide
FBS	fetal bovine serum
GABA	gamma-aminobutyric acid
*GDF15*	*Growth Differentiation Factor 15*
GSEA	gene set enrichment analysis
HaCaT	human adult low calcium temperature keratinocytes
*HMOX1*	*Heme Oxygenase 1*
*HAS3*	*hyaluronan synthase-3*
Hs68	human foreskin fibroblasts
*IFITM1*	*Interferon-Induced Transmembrane Protein 1*
*IL6*	*Interleukin 6*
iNOS	inducible NO synthase
*KF*	*Kochiae Fructus*
LC-QTOF-MS	Liquid Chromatography—Quadrupole Time-of-Flight—Mass Spectrometry
LPS	lipopolysaccharide
NC	untreated control
NO	nitric oxide
*MMP-1*	*matrix metalloproteinase-1*
*MMP-13*	*matrix metalloproteinase-13*
MTT	3-(4,5-dimethyl-2-thiazolyl)-2,5-diphenyl-2H-tetrazolium bromide
PABA	4-aminobenzoic acid
PGE2	prostaglandin E2
poly I:C	polyinosinic-polycytidylic acid
qPCR	quantitative real-time PCR
RA	retinoic acid
TGF-β	transforming growth factor-beta
TLR3	toll-like receptor 3
TNF- α	tumor necrosis factor-α
UV	ultraviolet
UVB	ultraviolet B
VEGF	vascular endothelial growth factor
WPBS	whole-plant *Bassia scoparia*
