# Supplementary material for: Multifunctional Dermatological Effects of Whole-Plant Bassia scoparia Extract: Skin Repair and Protection
Source: Curr Issues Mol Biol. 2025 Aug 4;47(8):617. doi: 10.3390/cimb47080617 (PMC12384579; doi:10.3390/cimb47080617)
Supplement: Supplementary file 1 [file cimb-47-00617-s001.zip › File S1. Transcriptomic analysis tools and scripts..docx]

**Software and Scripts for Generating Transcriptomic Outputs**

1. **FastQC (v.0.11.9)**

**for file in /path/to/raw_fastq/*.fastq.gz; do**

**fastqc -o /path/to/fastqc_output "$file"**

**done**

1. **Cutadapt Tool (v.4.6)**

***# Cutadapt trimming for paired-end reads (Cutadapt v4.6)***

**for file1 in /path/*_1.fastq.gz; do**

**# Find the paired file (_2.fastq.gz)**

**file2="${file1/_1.fastq.gz/_2.fastq.gz}"**

***# Define output file paths***

**output_file1="/path/$(basename $file1)"**

**output_file2="/path/$(basename $file2)"**

***# Run Cutadapt***

**cutadapt -a GATCGGAAGAGCACACGTCTGAACTCCAGTCAC \**

**-A GATCGGAAGAGCACACGTCTGAACTCCAGTCAC \**

**-o "$output_file1" -p "$output_file2" "$file1" "$file2"**

**Done**

1. **Hisat2 Tool (v2.2.1)**

***# HISAT2 alignment for paired-end reads (HISAT2 v2.2.1, samtools v1.15)***

**for sample in /path/to/trimmed_reads/*_1.fastq.gz; do**

**sample_id=$(basename "$sample" "_1.fastq.gz")**

**file1="/path/to/trimmed_reads/${sample_id}_1.fastq.gz"**

**file2="/path/to/trimmed_reads/${sample_id}_2.fastq.gz"**

**output_log="/path/to/hisat2_output/${sample_id}.log"**

**output_bam="/path/to/hisat2_output/${sample_id}.bam"**

**./hisat2 -p 8 --rna-strandness RF \**

**-x /path/to/genome_index/genome_reference_hg38 \**

**-1 "$file1" -2 "$file2" 2> "$output_log" | \**

**samtools view -bS -o "$output_bam"**

**done**

1. **featureCounts Tool (v2.0.8)**

***# Gene-level read quantification using featureCounts (v2.0.8)***

**./featureCounts \**

**-T 10 \ *# Number of threads***

**-p \ *# Paired-end reads***

**-s 2 \ *# Strand-specific protocol* (reverse)**

**-t exon \ *# Feature type: exon***

**-g gene_id \ *# Attribute type: gene_id***

**-a /path/to/annotation/Homo_sapiens.GRCh38.113.gtf \**

**-o /path/to/output/CountMatrix.txt \**

**/path/to/bam_files/*.bam**
