# Supplementary figures and images for: Multifunctional Dermatological Effects of Whole-Plant Bassia scoparia Extract: Skin Repair and Protection
Source: Curr Issues Mol Biol. 2025 Aug 4;47(8):617. doi: 10.3390/cimb47080617 (PMC12384579; doi:10.3390/cimb47080617)

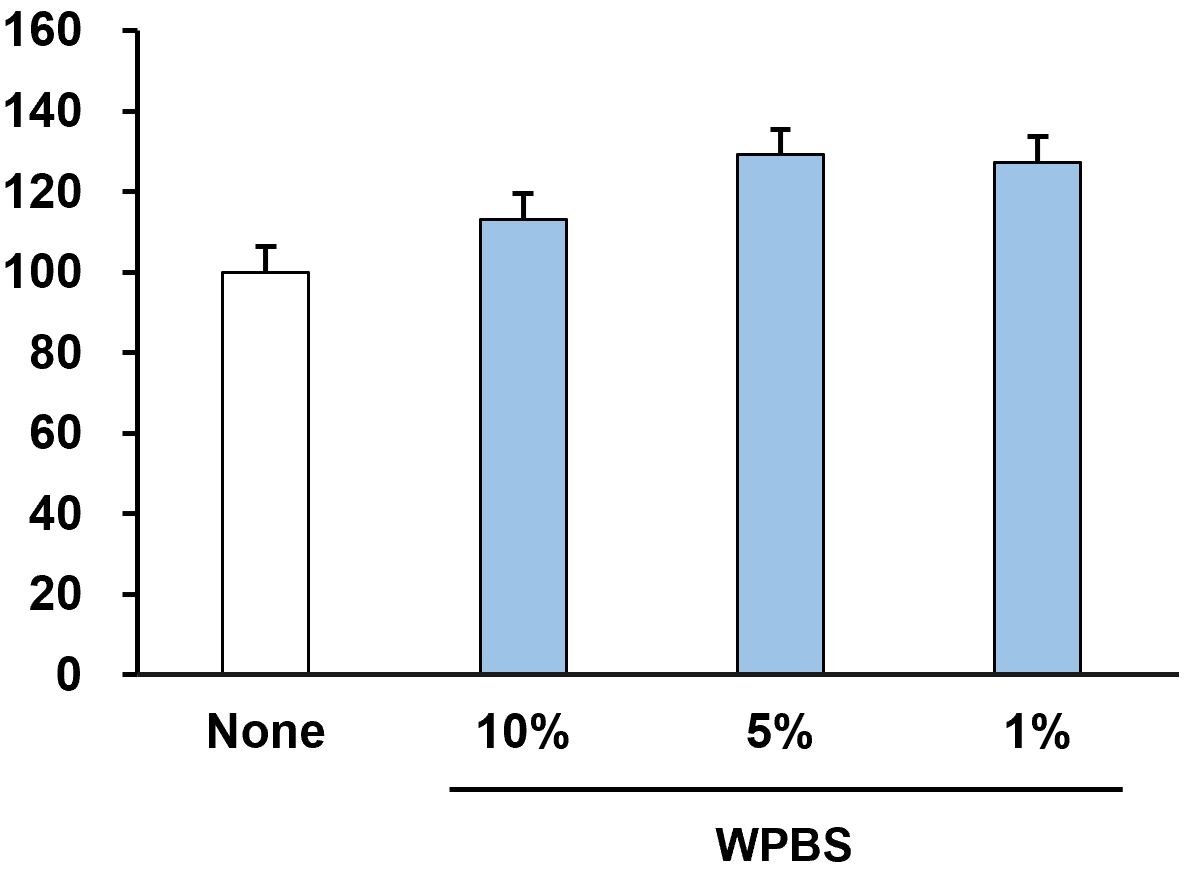

Supplement: Supplementary file 1 [file cimb-47-00617-s001.zip › Figure S1. Effect of WPBS on HaCaT cell viability..tif]

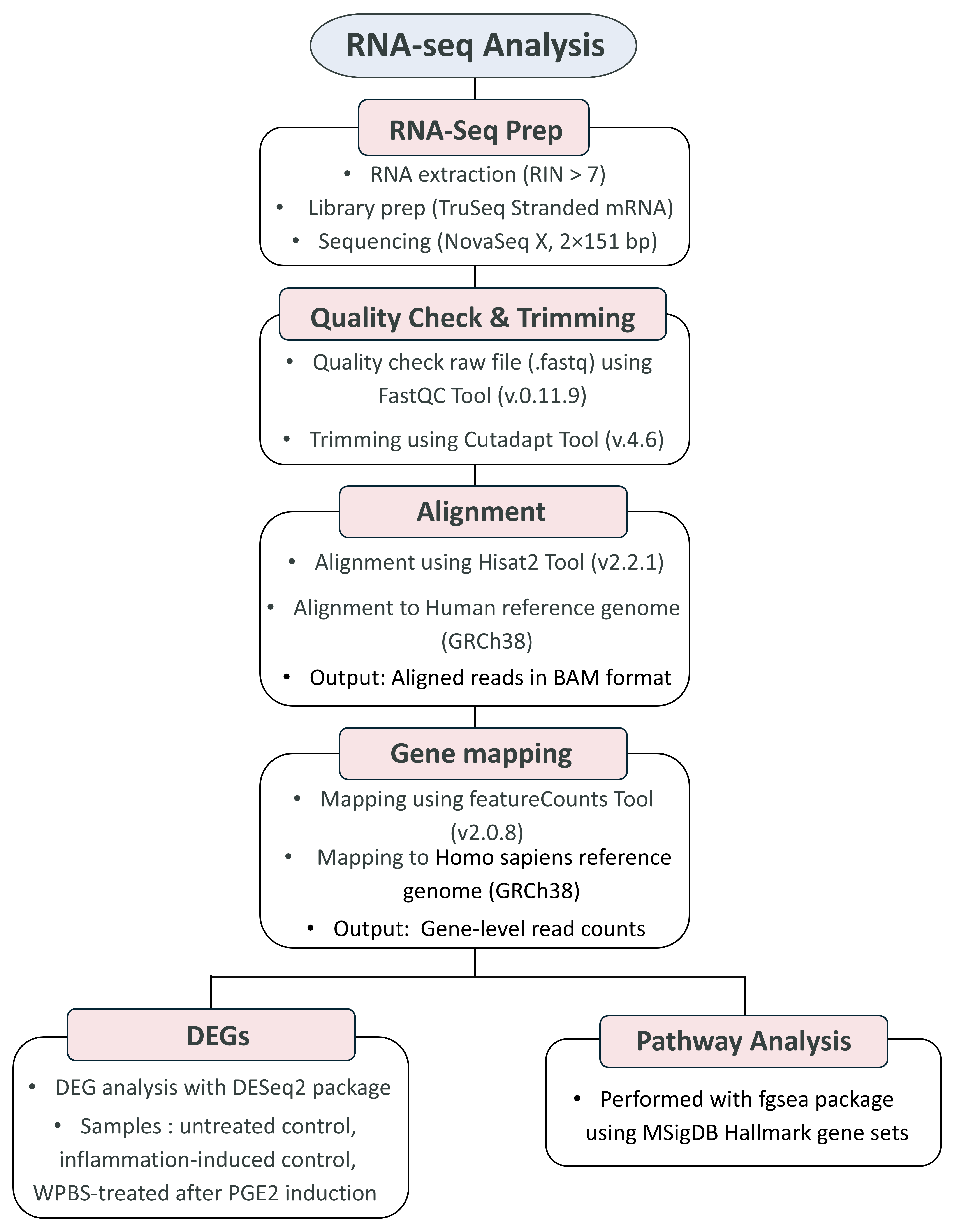

Supplement: Supplementary file 1 [file cimb-47-00617-s001.zip › Figure S2. Flowchart for bioinformatic analysis.tif]
